# Comparative Genomic Analysis Reveals Genetic Mechanisms of the Variety of Pathogenicity, Antibiotic Resistance, and Environmental Adaptation of *Providencia* Genus

**DOI:** 10.3389/fmicb.2020.572642

**Published:** 2020-10-27

**Authors:** Chao Yuan, Yi Wei, Si Zhang, Juan Cheng, Xiaolei Cheng, Chengqian Qian, Yuhui Wang, Yang Zhang, Zhiqiu Yin, Hong Chen

**Affiliations:** ^1^Department of Sanitary Toxicology and Chemistry, School of Public Health, Tianjin Medical University, Tianjin, China; ^2^Tianjin Key Laboratory of Environment Nutrition and Public Health, Tianjin Medical University, Tianjin, China; ^3^Center for International Collaborative Research on Environment Nutrition and Public Health, Tianjin Medical University, Tianjin, China; ^4^Key Laboratory of Molecular Microbiology & Technology, Ministry of Education, Tianjin Economic-Technological Development Area, Tianjin, China; ^5^Center for Microbial Functional Genomics and Detection Technology, Ministry of Education, Nankai University, Tianjin, China; ^6^Department of Dermatology, Tianjin Union Medical Center, Tianjin, China; ^7^College of Life Science, Nankai University, Tianjin, China; ^8^National Engineering Laboratory for Efficient Utilization of Soil and Fertilizer Resources, College of Resources and Environment, Shandong Agricultural University, Tai’an, China

**Keywords:** *Providencia*, comparative genomics, pan-genome, pathogenicity, antibiotic resistance, environmental adaptation

## Abstract

The bacterial genus *Providencia* is Gram-negative opportunistic pathogens, which have been isolated from a variety of environments and organisms, ranging from humans to animals. *Providencia alcalifaciens*, *Providencia rettgeri*, and *Providencia stuartii* are the most common clinical isolates, however, these three species differ in their pathogenicity, antibiotic resistance and environmental adaptation. Genomes of 91 isolates of the genus *Providencia* were investigated to clarify their genetic diversity, focusing on virulence factors, antibiotic resistance genes, and environmental adaptation genes. Our study revealed an open pan-genome for the genus *Providencia* containing 14,720 gene families. Species of the genus *Providencia* exhibited different functional constraints, with the core genes, accessory genes, and unique genes. A maximum-likelihood phylogeny reconstructed with concatenated single-copy core genes classified all *Providencia* isolates into 11 distant groups. Comprehensive and systematic comparative genomic analyses revealed that specific distributions of virulence genes, which were highly homologous to virulence genes of the genus *Proteus*, contributed to diversity in pathogenicity of *Providencia alcalifaciens*, *Providencia rettgeri*, and *Providencia stuartii*. Furthermore, multidrug resistance (MDR) phenotypes of isolates of *Providencia rettgeri* and *Providencia stuartii* were predominantly due to resistance genes from class 1 and 2 integrons. In addition, *Providencia rettgeri* and *Providencia stuartii* harbored more genes related to material transport and energy metabolism, which conferred a stronger ability to adapt to diverse environments. Overall, our study provided valuable insights into the genetic diversity and functional features of the genus *Providencia*, and revealed genetic mechanisms underlying diversity in pathogenicity, antibiotic resistance and environmental adaptation of members of this genus.

## Introduction

Bacteria of the genus *Providencia* are Gram-negative opportunistic pathogens, belonging to the *Proteae* in the *Enterobacteriaceae* family. *Providencia* could be isolated from a variety of environments and organisms (O’Hara et al., 2000; Foti et al., 2009; Interaminense et al., 2010). *Providencia* isolated from human stool samples are often thought of as part of the natural human gut flora or the cause of gastric disturbances (“travelers’ diarrhea”) (Ryan et al., 1992; Sobreira et al., 2001). According to previous reports, *Providencia* could sub-divided into 10 recognized species, *Providencia alcalifaciens*, *Providencia burhodogranariea*, *Providencia heimbachae*, *Providencia rettgeri*, *Providencia rustigianii*, *Providencia sneebia*, *Providencia stuartii*, *Providencia thailandensis*, *Providencia vermicola*, and *Providencia huaxiensis* (Penner and Hennessy, 1979; Hickman-Brenner et al., 1983; Yoh et al., 2005; Somvanshi et al., 2006; Juneja and Lazzaro, 2009; Khunthongpan et al., 2013; Jneid et al., 2016; Ksentini et al., 2019).

The species *Providencia alcalifaciens*, *Providencia rettgeri*, and *Providencia stuartii* are the most common clinical isolates, causing urinary tract and other nosocomial infections in humans (O’Hara et al., 2000; Yoh et al., 2005). These three species differ in their pathogenicity. *Providencia stuartii* is the species most frequently attributed to urinary tract infections (UTI) in patients of advanced ages (Mobley et al., 1986; Johnson et al., 1987; Kurmasheva et al., 2018). Some strains of *Providencia alcalifaciens*, but no strains of *Providencia rettgeri* or *Providencia stuartii*, are invasive in human cell lines (Albert et al., 1995; Janda et al., 1998; Sobreira et al., 2001; Maszewska et al., 2010). Strains of *Providencia alcalifaciens* isolated from diarrheal samples are able to invade several cultured mammalian cells *in vitro*, e.g., INT-407, HEp-2, HeLa, Vero, and Caco-2 (Guth and Perrella, 1996; Magalhaes et al., 1996; Janda et al., 1998; Murata et al., 2001; Maszewska et al., 2010). The pathogenicity of *Providencia rettgeri* against medflys, determined by assessing the effect of the bacteria on medfly egg hatching and development, has also been reported (Msaad Guerfali et al., 2018).

Species of the genus *Providencia* have intrinsic resistance to the antibiotics colistin and tigecycline, and bacterial strains with this resistance phenotype are often referred to as a multidrug-resistant (MDR) (Abdallah and Balshi, 2018). Among *Providencia* species, *Providencia rettgeri* and *Providencia stuartii* are the most common clinical MDR species of the genus *Providencia*, and are known to be the primary cause of several nosocomial outbreaks (Tumbarello et al., 2004; Aubert et al., 2005). MDR *Providencia rettgeri* and *Providencia stuartii*, especially integron-mediated MDR strains, have been isolated from some place of world (Mahrouki et al., 2014; Giakkoupi et al., 2015; Shin et al., 2018; Mbelle et al., 2020). Integrons are genetic elements that allow efficient capture and expression of exogenous genes. Integrons are recognized for their role in the dissemination of antibiotic resistance, particularly among Gram-negative bacterial pathogens (Gillings et al., 2008; Gillings, 2014). However, the role of integrons in the acquisition of drug resistance in *Providencia* is not yet known.

Horizontal gene transfer (HGT) has remained the key driver of bacterial evolution by allowing bacteria to rapidly acquire intricate new traits such as virulence and antibiotic resistance (AR) genes with the help of mobile genetic elements (MGEs) (Zhong et al., 2019). In this study, a comprehensive comparative genomics analysis was performed on the genus *Providencia* to investigate the distribution of virulence genes, AR genes and some special environmental adaptation genes. From a comprehensive perspective, our findings reveal the genetic mechanisms underlying diversity of pathogencity and antibiotic resistance of *Providencia*.

## Materials and Methods

### Bacterial Strains and DNA Extraction

Bacterial strains in this research are listed in Supplementary Table S1. The 28 *Providencia* strains sequenced in this research were obtained from the Polish Collection of Microorganisms (PCM) at the Hirszfeld Institute of Immunology and Experimental Therapy, Polish Academy of Sciences (Wrocław, Poland). All strains were stored at −80°C in Luria-Bertani (LB) broth supplemented with 20% (v/v) glycerol and cultured at 37°C in LB broth. Bacteria Extraction Kit (CWBIO Co., Ltd., China) for DNA extractions from each strain was used according to the manufacturer’s instructions. The sequencing data is available on NCBI GenBank database under project PRJNA580371.

### Genome Sequencing and Raw Data Processing

The genomic sequencing performed using Solexa pair-end sequencing technology (Illumina, Little Chesterford, Essex), with a depth of 90–100-fold coverage. The reads were *de novo* assembled using Velvet Optimiser v2.2 (Zerbino and Birney, 2008). The assembly statistics for all newly sequenced *Providencia* genomes were showed in Supplementary Table S1. The annotation of newly sequenced genomes was performed by NCBI Prokaryotic Genome Annotation Pipeline^1^. All genomes data in our research were accessed by CheckM (Parks et al., 2015), and related statistics were showed in Supplementary Table S1.

### Phylogenetic Analysis Based on Single-Copy Core Genome

Orthologous groups were delimited using OrthoFinder (Emms and Kelly, 2015) with default parameter (for BLASTp: outfmt = 6, evaule = 0.001; for MCL: I = 1.5). The single-copy core gene families, core gene families, and pan-gene families were extracted based on the OrthoFinder output results. Nucleotide sequences of the single-copy core gene families were extracted and then aligned using MAFFT (Katoh and Standley, 2013). The phylogenetic analysis of *Providencia* was performed using the single-nucleotide polymorphisms (SNPs) set present in 371 single-copy core gene families. It was considered that homologous recombination could occur in bacterial genome and can confound the phylogenetic analysis. We identified and removed the putative recombinational regions of SNPs, using ClonalFrameML software (Didelot and Wilson, 2015). The Maximum Likelihood (ML) tree was constructed using MEGA 7 software (Kumar et al., 2016) [with the General Time Reversible (GTR) model].

### Core and Pan-Genome Analysis

The pan-genome analysis was performed based on the Heap’s law for pan-genome models (Tettelin et al., 2008). The total number of gene families (*n*, y axis) for increasing values of the number genomes (*N, x*-axis) is shown. The curve was a least-squares fit based on the power law (*n* = :*N*^γ^) to the averages. The core genome analysis was performed by regression analysis (Bottacini et al., 2010). A weighted least square regression by fitting the power law *n* = *ê*exp(*m* × *N*) + Θ (*N*: the number of genomes, *n*: the number of core gene families, Θ: a constant value representing the predicted minimum number of core genes, *ê* and *m*: parameters).

### Genetic Population Structure Analysis and Gene Functional Category

The average nucleotide identity (ANI) were calculated using the JSpecies 1.2.1 software (Richter and Rosselló-Móra, 2009). Population structure analysis was conducted using the Bayesian Analysis of Population Structure (BAPS) program with initial K value (7,7,8,8,9,9,9,10,10,10,11, 11,11,12,12,13,13) (Cheng et al., 2013). We analyzed the functional category of the gene family based on Cluster of Orthologous Groups (COG) assignment. The functional annotation of proteins was performed by alignment against the COG database from NCBI using BLASTp with *E*-value of 1e-5. The PHAge Search Tool Enhanced Release (PHASTER) was utilized to find the prophages (Arndt et al., 2016). Genomic islands were predicted using the IslandViewer 4 database (Bertelli et al., 2017). The clustered regularly interspaced short palindromic repeats (CRISPRs) were predicted using the CRISPR recognition tool (CRT1.2) with default parameters (Bland et al., 2007).

### Identification Virulence Genes and Resistance Genes Identification

To identity the virulence genes and resistance genes, protein sequences of all *Providencia* genomes were aligned using BLASTp with an *E*-value cutoff < 1e-6, identity > 60%, and coverage > 60% against the dataset from Virulence Factors Database (VFDB) (Liu et al., 2019), and Comprehensive Antibiotic Database (CARD) (Jia et al., 2017). To examine the virulence-related elements, we screened gene clusters using the LS-BSR tool (Sahl et al., 2014). These results were visualized using the pheatmap R packages.

### Identification of Macromolecular Secretion Systems

To detected and visualized the macromolecular systems in the *Providencia* genus, we used the programs MacSyFinder and TXSScan (Abby and Rocha, 2017) with default parameters. Furthermore, the type VI secretion system (T6SS) was identified and analyzed based on previous research (Yuan et al., 2019) and combined with the results from SecReT6 (Li et al., 2015) with default parameters.

### Group-Specific Core Genome Analysis

To examine the Group-specific core genomes, we constructed a pan-genome-cluster map of the gene families across all 91 *Providencia* genomes. Results was visualized using the R command from the pheatmap package.

## Results and Discussion

### Pan-Genome Analysis

To characterize the genomic composition among *Providencia*, 28 genomes from clinical isolates were sequenced for pan-genome analysis and comparative genomic analysis. Combining with sequencing data of 63 published *Providencia* genomes from National Center for Biotechnology Information (NCBI) databases, pan-genome analysis was performed (Supplementary Table S1). The principle for our data-selection was that a *Providencia* strain with full type of genome files available on NCBI at the beginning of our project, thus 3 species were not selected in our research, including *Providencia vermicola*, *Providencia thailandensis* and *Providencia hauxiensis*. The 91 genomes contained 14,720 orthologous groups (defined as gene families) that were classified into three classes: core, accessory, and unique genes. 716 gene families (4.9%) shared by all strains formed the core genome, while remaining 14,004 gene families (95.1%) formed variable genome. This variable genome of 91 isolates of *Providencia* constituted a substantial portion (95.1%) of the pan-genome of the genus, indicating a high degree of genetic variation. Among these variable gene families, 5137 gene families formed the unique genome (specific to a single strain), and 8867 gene families formed the accessory genome (present in more than one strain).

**FIGURE 1:**
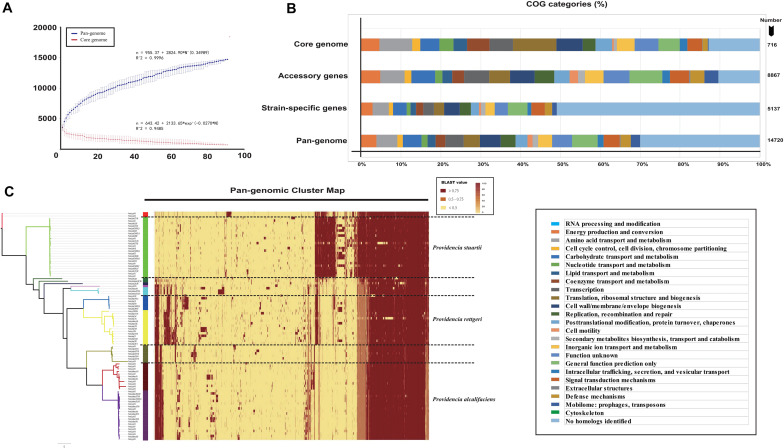
Genetic diversity of genome in *Providencia.* **(A)** Core and pan-genome curves showed the downward trend of the core gene families and the upward trend of the pan-gene families with the increase in the number of genomes. The error bars indicated the standard deviation of the number of core and pan-gene families. The fitting mathematical functions of the core and pan-genome curves were shown. **(B)** Distribution of COG categories for each gene family set. **(C)** Cluster map of the pan genome of *Providencia*.

To further analyze the core and pan-genomes, we characterized the core and pan-genomes of *Providencia* based on Heap’s law pan-genome model (Figure 1A). The pan-genomes of 91 *Providencia* genomes showed a clear linear upward trend based on Heap’s law pan-genome model, and a robust fit to the data obtained with an increasing power law with γ = 0.34989 (Figure 1A). The exponent γ > 0 represented that *Providencia* was an open pan-genome species. An open pan-genome helps bacteria respond to diverse environments. We analyzed the functional category of the *Providencia* pan-genome by searching the Clusters of Orthologous Groups (COG) database (Figure 1B and Supplementary Table S2). Genes in the categories “no homologs identified” and “function unknown” formed a large proportion (43.5%) of the pan-genome. In the unique gene groups, the proportion of genes in these categories could be up to 60%, whereas the proportion was no more than 15% in the core and accessory gene groups. These findings revealed that unique genes conferred a variety of functions to *Providencia*, which was probably related to the environmental response of the bacteria. Cluster map of the pan genome of *Providencia* was draw (Figure 1C) for Group-specific gene analysis. There were large number of uncharacterized unique genes in the pan-genome of *Providencia* needed further attention.

### Phylogenetic Analysis of *Providencia*

**FIGURE 2:**
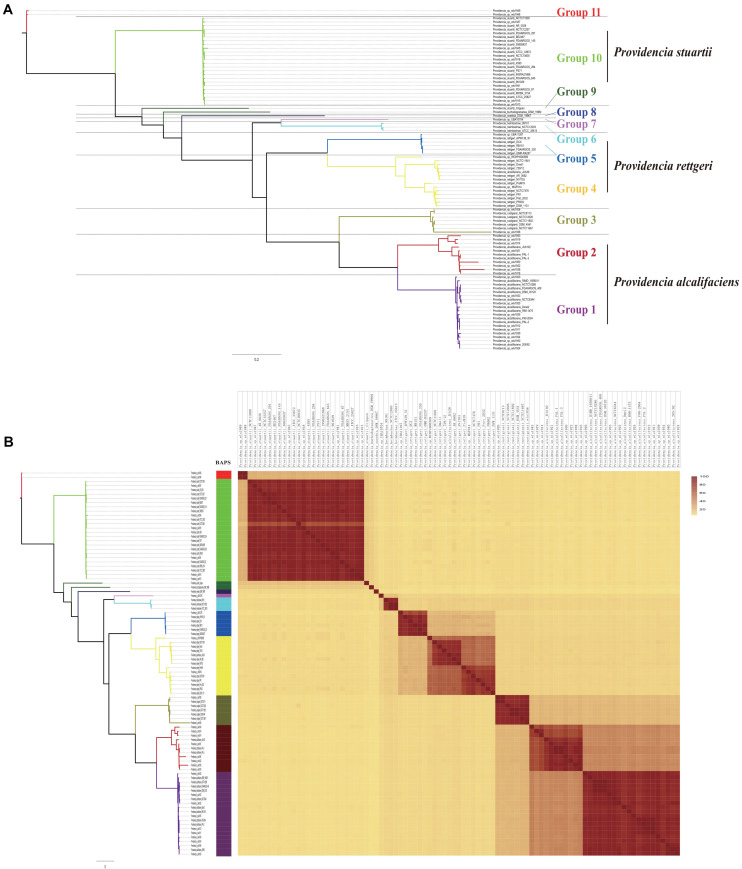
Phylogenetic analysis and whole genome nucleotide identity. **(A)** ML phylogeny was constructed based on SNPs across 372 single-copy core gene families shared by the 91 *Providencia* genomes. **(B)** The heatmap presents the average nucleotide identity.

To assess the phylogenetic relationship of the genus *Providencia*, a maximum-likelihood phylogenetic tree was reconstructed using 371 concatenated single-copy core genes from 28 newly sequenced and 63 published *Providencia* strains. The resulting showed a reliable delineation of phylogenetic relationships across the genus. According to the topological structure, the 91 strains were divided into 11 lineages (Figure 2A). Genomic similarities among strains were further explored by genetic population structure analysis using Bayesian analysis of population structure (BAPS) and calculation of the ANI value to estimate genetic distance between strains at the genomic level (Figure 2B). Genetic population structure analysis divided the *Providencia* genus into 11 groups (Groups 1–11), which corresponded to the phylogenetic tree. *Providencia stuartii* was mainly distributed in Group 10, while *Providencia rettgeri* was distributed in Groups 4 and 5. Although Group 2 contained some species-unknown strains, it partly contained *Providencia alcalifaciens*, which revealed a closed relationship. Phylogenetic relationships of strains observed by reconstructing phylogenetic trees based on concatenated core genes provide a high resolution and can reveal the presence of different intrageneric complexes. Thus, as in Figure 2A, both *Providencia alcalifaciens* and *Providencia rettgeri* had a new subgroup which was in agreement with the classification of strains into a new Group after population genetic analysis, which indicated that genus *Providencia* needed to be reorganized in the future. Location of *Providencia alcalifaciens* JUb39 in Group 4, which only contained isolates of *Providencia rettgeri*, probably reflected an error in species identification. *Providencia* sp. wls1948 and *Providencia* sp. wls1949 in Group 11 diverged independently from other members, and this was the first or most ancient divergence of the 91 *Providencia* strains examined in this study. For further detailed comparative genomics analysis of the genus *Providencia*, we focused on Groups 4 and 5 (*Providencia rettgeri*), Group 10 (*Providencia stuartii*), and Groups 1 and 2 (*Providencia alcalifaciens*).

### Antimicrobial Genotypic Profiles in *Providencia*

**FIGURE 3:**
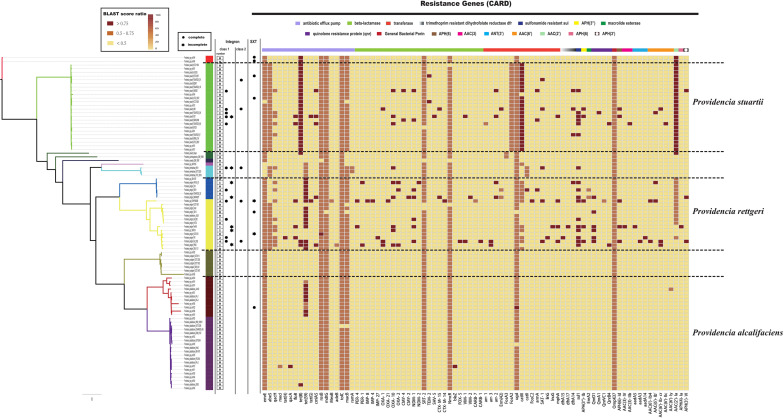
The genotypic profiles of resistance genes across all 91 *Providencia*. Color coding for resistance genes corresponds to the score ratio recorded for each genome when screened with the reference The Comprehensive Antibiotic Resistance Database (CARD).

Clinically, species of the genus *Providencia* are often referred to as MDR, especially *Providencia rettgeri* and *Providencia stuartii*, which have been reported to cause nosocomial outbreaks. In contrast, rarely case about MDR *Providencia alcalifaciens* was reported. Therefore, the distribution of resistance genes in the genus *Providencia* was investigated (Figure 3 and Supplementary Table S3). Almost all species had genes encoding different types of antibiotic efflux pumps, including resistance-nodulation-cell division (RND) types, major-facilitator superfamily (MFS) types, and ATP-binding cassette (ABC) types, which mainly confer resistance to peptide antibiotics and macrolide antibiotics. Furthermore, almost all species contained genes encoding different types of beta-lactamase and *pmr* phosphoethanolamine transferase, which confer resistance to cephalosporin, carbapenem, cephamycin, penam, monobactam, and peptide antibiotics. The genes ARO:3000166 and ARO:3002685, encoding a MFS antibiotic efflux pump and chloramphenicol acetyltransferase (CAT), respectively, were mainly distributed in Group 10 (*Providencia stuartii*). ARO:3004441, a gene encoding a MFS antibiotic efflux pump, was mainly distributed in Groups 2, 4, 5, 6, and 7. Group 2 only contained isolates of *Providencia alcalifaciens* and Groups 4 and 5 was a *Providencia rettgeri*-specific group. The gene ARO:3002523, encoding resistance to aminoglycoside antibiotics, was mainly distributed in Groups 4, 5, 10, and 11. There were also many sporadically distributed-resistance (SDR) genes distributed in Groups 4, 5, and 10 (*Providencia rettgeri* and *Providencia stuartii*). This could explain why *Providencia rettgeri* and *Providencia stuartii* were considered less susceptible to several antibiotics in clinical.

**FIGURE 4:**
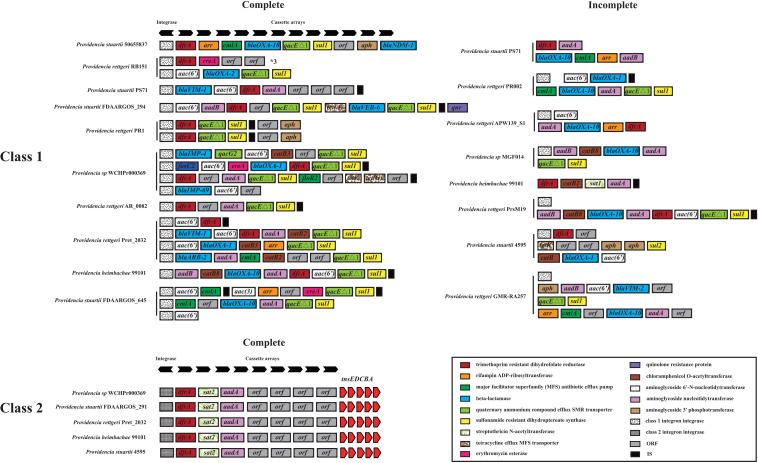
The genetic organization of integrons in 91 *Providencia*. The same gene species are shown in the same color. The black arrows represent the direction of gene transcription.

**FIGURE 5:**
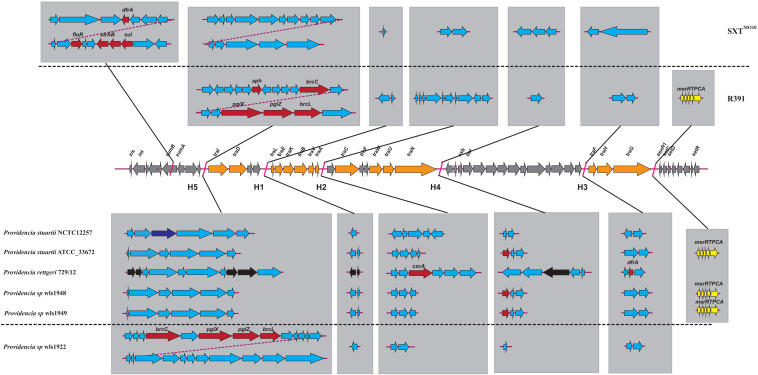
The genetic structure of SXT elements in *Providencia*. The middle line demonstrated the backbone of core genes in SXT ICEs. The genetic structure of SXT^*MO*10^ and R391 were shown above the middle line. Orange arrows indicated the coding genes involved in conjugative transfer process. Gray arrows indicated other genes in conserved regions. The inserted genes of SXT elements in five hotspots (HS1–5) and other variable regions were shown in light gray frame. Red arrows indicated antibiotic resistance genes. Dark blue arrows indicated restriction-modification system. Yellow arrows indicated heavy metals resistance genes. Black arrows indicated pseudogenes. Light blue arrows indicated other genes in hotspots and variable regions.

We hypothesized that SDR genes distributed in *Providencia rettgeri* and *Providencia stuartii* were mainly acquired by HGT, especially from integrons. Integrons play a key role in the dissemination of antibiotic resistance, particularly among Gram-negative bacterial pathogens. Previous studies had shown that some genomes of *Providencia rettgeri* and *Providencia stuartii* harbored integrons, which contributed to MDR (Mahrouki et al., 2014; Giakkoupi et al., 2015; Mbelle et al., 2020). To prove our hypothesis, we determined the distribution of integrons in *Providencia*. Genomes of members of the genus *Providencia* contained integrons and the SXT element. According to previous reports, Integrons could be classified and divided into four groups, termed class 1–4 integrons. Known as multi-resistant integrons (RIs), class 1–3 integrons are more commonly detected in Gram-negative microorganisms. Class 4 integrons were first identified on the SXT element of *Vibrio cholera* (Gillings, 2014; Deng et al., 2015). Based on the differences and divergence in the sequences of integrase from integrons and the SXT element, the integrons and SXT element identified in this study mainly included class 1 integrons, class 2 integrons, and the SXT element (Figure 3). In the genus *Providencia*, strains with integrons and the SXT element were mainly distributed in Groups 4 and 5 (*Providencia rettgeri*) and Group 10 (*Providencia stuartii*). In addition, some strains contained multiple class 1 integrons or even two classes of integrons. Further analysis of the relationship between the distribution of integrons and SXT element and SDR genes in *Providencia* focused on the structure of the integrons and SXT element in *Providencia* (Figures 4, 5). *Providencia* contained more class 1 integrons than class 2 integrons. Moreover, the class 1 integrons contributed a variety of cassette arrays that contained multiple resistance genes. In contrast, class 2 integron structures were conserved with low diversity of cassette genes. The type of resistance genes within integrons corresponded to the type of SDR genes in *Providencia*. Therefore, integrons are one of the main sources of multidrug resistance in *Providencia*. The SXT element was reported as a source of resistance genes in *Vibrio cholera* (Hochhut et al., 2001). However, in *Providencia*, the SXT element contained very few resistance genes that could contribute to multidrug resistance. As shown in Figure 5, none of the strains containing SXT elements harbored SDR genes. Overall, except for some resistance genes shared by all strains, the genus *Providencia* contained SDR genes that could caused a MDR phenotype of *Providencia rettgeri* and *Providencia stuartii*. This could explain why *Providencia rettgeri* and *Providencia stuartii* were considered less susceptible to several antibiotics in clinical settings. These SDR genes were mainly derived from class 1 integrons and class 2 integrons rather than the SXT element.

### Virulence Genotypic Profiles Revealed the Pathogenicity of *Providencia*

**FIGURE 6:**
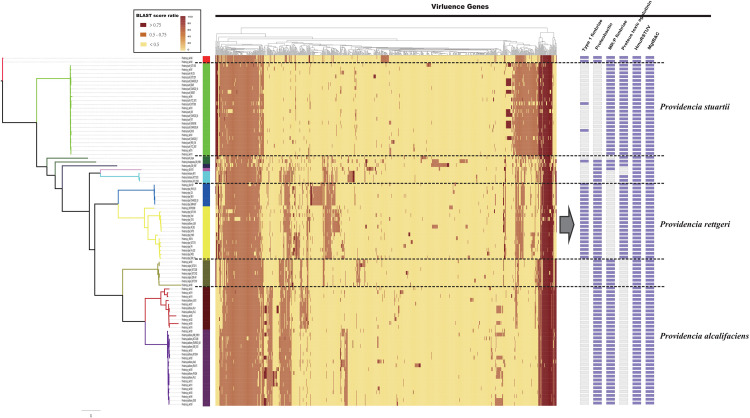
The genotypic profiles of virulence genes across all 91 *Providencia*. Color coding for virulence genes corresponds to the score ratio recorded for each genome when screened with the reference Virulence Factors Database (VFDB). The right side of the gray arrows represents the result of cluster analysis.

The distribution of virulence genes in *Providencia* was investigated to identify key pathogenicity genes of *Providencia rettgeri, Providencia stuartii*, and *Providencia alcalifaciens.* All 91 *Providencia* genomes were locally aligned against the Virulence Factors Database (Liu et al., 2019) to identify virulence genes, cluster map was shown in Figure 6 (Supplementary Table S3). The virulence genes shared by all strains were associated with flagella biosynthesis, LPS (lipopolysaccharide), and *ompA*. The genes *mgtB* and *mgtC*, which encode an Mg^2+^ uptake system in *Salmonella enterica* serovar Typhimurium, were distributed in almost all *Providencia* strains. MgtB and MgtC are key virulence factors of *Salmonella enterica* serovar Typhimurium, with MgtC required for survival inside macrophages and MgtB involved in transporting Mg^2+^ from the periplasm to the cytoplasm (Perez et al., 2009; Lee and Groisman, 2012; Choi et al., 2017). *phoP/Q* was also distributed in almost all *Providencia* strains, and this gene pair has been reported to regulate the expression of *mgtB* and *mgtC* in *Salmonella enterica* serovar Typhimurium (Choi et al., 2017). Almost all *Providencia* strains harbored genes encoding a direct heme uptake system (*hmuRSTUV*). This system allows *Proteus mirabilis* to uptake and utilize hemin and hemoproteins as iron sources. Furthermore, this system contributed to the pathogenicity of *Proteus mirabilis* in UTIs (Lima et al., 2007; Rocha et al., 2007; Schwiesow et al., 2018). These genes could account for the basic virulence of species of the genus *Providencia* during infection.

There were some virulence genes specifically distributed in *Providencia* strains. *Providencia alcalifaciens* (strains in Groups 1 and 2) contained genes encoding CsrA and Proteus toxic agglutinin. CsrA, as a global regulator in many bacteria, is usually critical for integrating the response to environmental cues with the regulation of important virulence genes (Mey et al., 2015; Hauslein et al., 2017; Ferreiro et al., 2018). Proteus toxic agglutinin, a surface-associated cytotoxin, was identified as another key virulence gene in *Proteus mirabilis* during UTIs (Mey et al., 2015; Hauslein et al., 2017; Ferreiro et al., 2018). Proteus toxic agglutinin, a surface-associated cytotoxin, was also key virulence genes in *Proteus mirabilis* during UTIs (Alamuri and Mobley, 2008; Alamuri et al., 2009). *Providencia rettgeri* (strains in Groups 4 and 5) had genes which encoded mannose-resistant *Proteus*-like fimbriae (MR/P), MR/P fimbria was well studied in many researches and contributed to virulence mediating adhesion to uroepithelial mucosal cells and to exfoliated urinary cells (Zunino et al., 2007). The presence of these genes in *Providencia rettgeri* implies that MR/P fimbriae could be very important for *Providencia rettgeri* in causing UTIs. In addition, genes encoding Type 1 fimbriae of uropathogenic *Escherichia coli* 536 were mainly distributed in Groups 4 and 5 (*Providencia rettgeri*). Type 1 fimbriae are the most common adhesins among both commensal and pathogenic isolates of *E. coli*, and their expression has been linked to successful establishment of UTIs and bacterial persistence in the urinary tract (Rocha et al., 2007). *Providencia stuartii* (strains in Group 10) contained genes encoding the proteobactin siderophore system. The proteobactin system has an iron acquisition function when the urinary tract is an iron-limiting environment (Alamuri et al., 2009; Himpsl et al., 2010). In summary, the differential distribution of virulence-related gene clusters can explain the differences in the pathogenicity of distinct isolates of *Providencia*.

### Macromolecular Secretion Systems Reflected the Pathogentic Potential of *Providencia*

**FIGURE 7:**
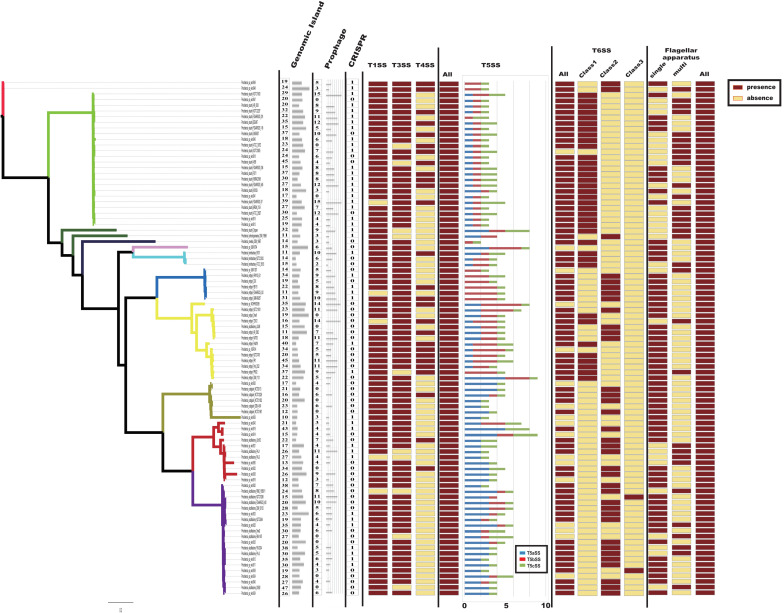
Macromolecular secretion systems distribution in *Providencia*. Dark red boxes represented the presence of a macromolecular system within a genome, while yellow boxes indicate the absence of a macromolecular system.

Bacteria usually interact with their surrounding environments by protein secretion (Abby and Rocha, 2017). In particular, pathogenic bacteria could secrete many virulence factors during colonization (Economou et al., 2006). In gram-negative bacteria, six types of secretion systems (T1SS to T6SS) have been identified and well characterized (Economou et al., 2006; Dalbey and Kuhn, 2012). To further analyze the virulence genes of *Providencia*, Macromolecular secretion systems in the 91 *Providencia* genomes were identified using the MacSyFinder (Figure 7). Flagellar apparatus and type V secretion system (T5SS) were restricted to the *Providencia* genus, while type I secretion system (T1SS), type III secretion system (T3SS) and type VI secretion system (T6SS) were distributed in almost all *Providencia* strains. Furthermore, numerous MGEs included clustered regularly interspaced short palindromic repeats (CRISPRs) (Supplementary Figure S1), genomic islands, and prophages that have been identified across the *Providencia* genomes (Figure 7). These elements were the major contributors to HGT, and drived the adaptation of bacteria to diverse niches.

Type IV secretion system (T4SS) could transport a diverse array of substrates into the host cells, and play fundamental roles in both pathogenesis and adaptation in the host cellular niche (Guglielmini et al., 2013). T4SS was mainly shared by strains in Groups 4 and 5 (*Providencia rettgeri*), Group 10 (*Providencia stuartii*) and Group 11, which were reported to predominantly cause UTIs in clinical settings. A maximum-likelihood phylogenetic tree was reconstructed based on core genes of T4SS in the genus *Providencia* (Supplementary Figure S2). Strains containing the SXT element clustered in the same lineage and there was discordance in the topology of the branching order and phylogenetic placement between this tree and the tree in Figure 2A, which revealed that T4SS in *Providencia* were acquired by HGT.

T3SS, common virulence-related secretory systems that evolved from the flagellum, allow the direct transfer of proteins from the bacterial cytosol into host cells. A maximum-likelihood phylogenetic tree was reconstructed based on core T3SS genes in *Providencia* and other reference bacteria including *Salmonella* Typhi CT18, *Salmonella* Typhimurium LT2, *Pseudomonas aeruginosa*, enterohemorrhagic *E. coli* (EHEC), *Yersinia pestis*, and *Shigella flexneri* (Supplementary Figure S3). Most phylogenies for core genes of T3SSs revealed evolutionary histories that were similar to those of concatenated single-copy core genes. Five strains of *Providencia alcalifaciens* were classified into the same lineage as *Salmonella*, while the remaining strains of *Providencia alcalifaciens* shared the same lineage as EHEC. T3SS in Salmonella helped bacteria to invade host cells (Lou et al., 2019), whereas in EHEC was only contributed to adhesion (Stevens and Frankel, 2014). This could explain why some isolates of *Providencia alcalifaciens* can invade cells.

**FIGURE 8:**
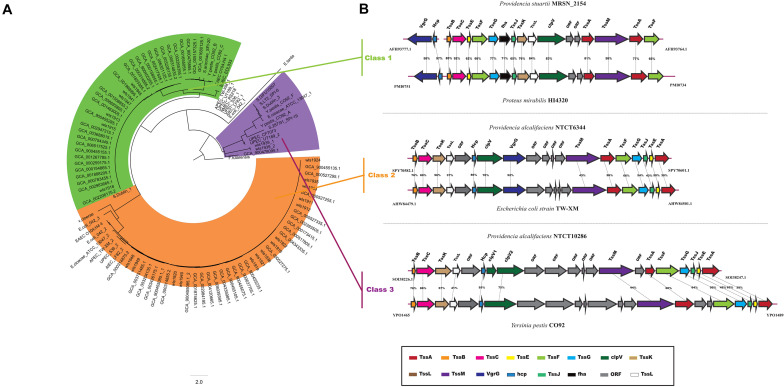
Model and phylogeny of T6SS in *Providencia*. **(A)** ML phylogeny generated from the shared TssF protein sequences in the *Providencia* T6SS. **(B)** The genetic organizations of T6SS. Homologous genes are shown in same color.

The T6SS is a multiprotein machinery, which is widespread in Gram-negative proteobacteria and has a variety of biological functions (Coulthurst, 2013; Zoued et al., 2014). In *E. coli*, T6SS have been categorized into three distinct groups: T6SS-1 to T6SS-3 (Russell et al., 2014). In our study, using the same method as in *E. coli*, a phylogenetic analysis of the *Providencia* genus and other classified strains including NMEC RS218, *Salmonella* LT2, EAEC 042, *Pseudomonas aeruginosa*, APEC TW-XN, *Edwardsiella tarda*, *Vibrio cholerae*, and *Francisella tularensis* was performed to classify the T6SSs into three classes (Figure 8A). The results suggested that T6SS gene clusters in the genus *Providencia* were probably acquired by HGT. The functions of three classes of T6SS are quite distinct: T6SS-1 often involved in biofilm formation; T6SS-2 are commonly involved in colonization, survival or invasion (human hosts); T6SS-3 major involved in antibacterial effectors. T6SSs were distributed in almost all *Providencia* strains. Strains in Group 10 (*Providencia stuartii*) all contained T6SS-1 genes, while strains in Group 1&2 (*Providencia alcalifaciens*) harbored genes encoding T6SS-2. In addition, strains in Group 4&5 (*Providencia rettgeri*) contained either T6SS-1 or T6SS-2. The genetic organization of three kinds of T6SS in *Providencia* was shown in Figure 8B. This species-specific distribution of T6SSs is a likely cause of the differences in pathogenicity of the three species of the genus *Providencia* that were the focus of this study (*Providencia stuartii*, *Providencia alcalifaciens*, and *Providencia rettgeri*). T6SS-2, which is related to invasion or survival in the host, was almost exclusively found within *Providencia alcalifaciens*. In contrast, the T6SS-1 in Groups 1 and 2 (*Providencia alcalifaciens*) and Groups 4 and 5 (*Providencia rettgeri*) enhances the resistance of bacteria against stress from the natural environment, which could promote the occurrence of HGT in the natural environment, especially in AR genes. Overall, the specific distribution of different types of T6SS contributes to diverse pathogenicity and adaptation of *Providencia*.

### Group-Specific Genes Revealed the Different Environmental Adaptation of *Providencia*

**FIGURE 9:**
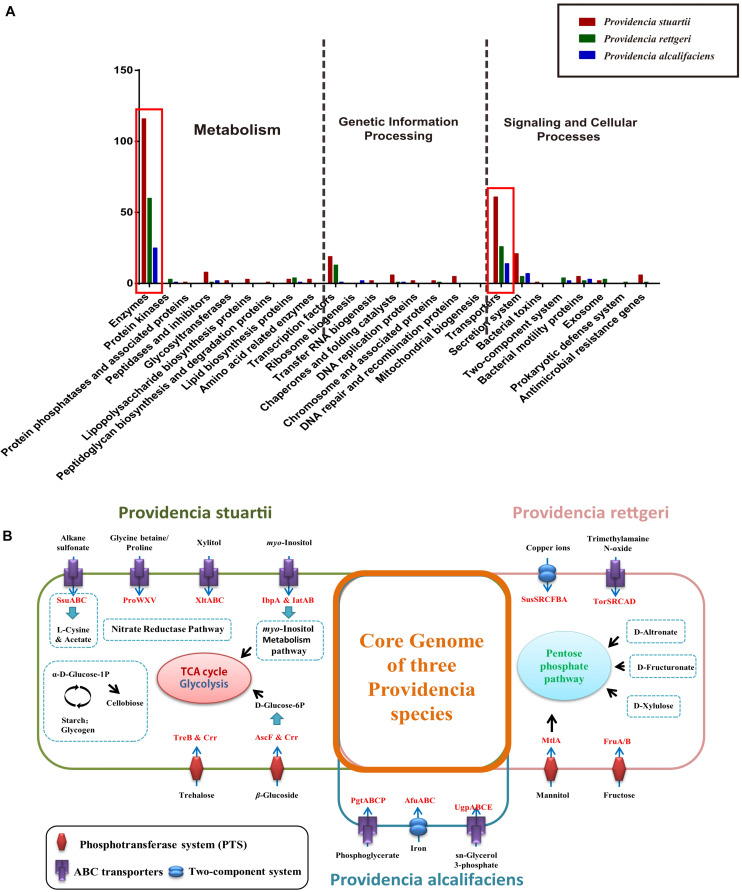
Functional enrichment of the species-specific core genomes of three *Providencia* species after KEGG annotation. **(A)** The detailed enrichment results of the species-specific core genomes after KEGG annotation. **(B)** The complete pathway modules of the species-specific core genomes of three *Providencia* species.

Group-specific gene analysis can reveal underlying profiles of gene families that are conserved among strains within a group, which may provide information not only about virulence of bacteria, but also about the metabolic features within the host environment. To identify group-specific gene families in the genus *Providencia*, an accessory genome was constructed by subtracting the core genome and low frequency genes from the pan-genome. For Groups 1 and 2 (*Providencia alcalifaciens*), Groups 4 and 5 (*Providencia rettgeri*), and Group 10 (*Providencia stuartii*), group-specific genes were extracted and identified (Supplementary Table S4). Based on KEGG annotation, gene function cluster analysis was assessed (Figure 9A). The functional categories “Enzymes in Metabolism” and “Transporters in Signaling and Cellular Processes” were enriched in all three groups of species-specific genes (red box in Figure 9A). These group-specific genes may be related to inherent differences in pathogenicity between the three species of *Providencia*. Through systemic analysis of these species-specific genes, the differential distribution of specific metabolic pathways and transport mechanisms in these three species was revealed (Figure 9B). In *Providencia alcalifaciens*, four ABC transporters for movement of alkane sulfonate, glycine betaine/proline, xylitol, and *myo*-inositol, respectively, two phosphotransferase systems (PTS) that transport trehalose and β-glucoside, respectively, nitrate reductase pathway; and *myo*-inositol metabolism pathway were identified. Compared to the other two species, *Providencia stuartii* contained more transporters and metabolic pathways that lead into the TCA cycle and glycolysis. In *Providencia rettgeri*, a two-component system for transport of copper ions, an ABC transporter for movement of trimethylamaine N-oxide, two PTS that transport mannitol and fructose, respectively, and some other pathways associated with carbohydrate metabolism that lead into the pentose phosphate pathway were identified. Strains of *Providencia stuartii* and *Providencia rettgeri* had more mechanisms of material transport and energy metabolism than strains of *Providencia alcalifaciens*, reflecting a stronger ability of the former two species to adapt to diverse environments. On the other hand, strains of *Providencia alcalifaciens* contained two ABC transporters (PtgABCP and UgpABCE) for transportation of phosphoglycerate and sn-glycerol 3-phosphate, respectively. Phosphoglycerate and *sn*-glycerol 3-phosphate were important metabolic intermediates in glycolysis, which usually maintained a certain concentration in eukaryotic cells. PtgABCP and UgpABCE for the acquisition of carbohydrates from nutrient poor environment likely contributed to invasion by *Providencia alcalifaciens*. Therefore, the group-specific metabolism-associated gene profiles of three species of the genus *Providencia* may reflect the specific nutrient niches in the different host environments and the mechanism in host-environment-adaption of pathogens.

## Conclusion

This study evaluated genetic diversity within the genus *Providencia* using comparative genomic analysis and pan-genome analysis, and elucidated virulence, antibiotic resistance, and host adaptation profiles of members of this genus. The pan-genome of the 91 *Providencia* strains was open and showed extensive genomic variability. Phylogenetic analysis divided the genus *Providencia* into 11 Groups and revealed the main distribution Groups of the three most common clinical species, *Providencia alcalifaciens* (Groups 1 and 2), *Providencia rettgeri* (Groups 4 and 5), and *Providencia stuartii* (Group 10). *Providencia* genomes exhibited high levels of evolutionary plasticity, with many gene families differing in size during evolution. HGT was the driver of genetic diversity that shaped genomes of members of the genus *Providencia*, which accounted for many of the genetic differences involved in virulence, antibiotic resistance, and environmental adaptation.

Our comparative genomic analysis revealed that strains of *Providencia* contained many virulence genes that were homologous to key virulence genes in *Proteus mirabilis*, such as *hmuRSTUV* and genes encoding proteus toxic agglutinin, MR/P fimbriae, and proteobactin. These virulence genes may contribute to the successful establishment of UTIs by *Providencia* in clinical settings. The distribution of virulence genes in *Providencia* was found to be Group-specific. Strains of Groups 4 and 5 (*Providencia rettgeri*) contained genes encoding T6SSs (class 1 or class 2), type 1 fimbriae, proteus toxic agglutinin, and proteobactin; strains of Groups 1 and 2 (*Providencia alcalifaciens*) harbored genes encoding T6SS-2, proteobactin, and MR/P fimbriae; and strains of Group 10 (*Providencia stuartii*) contained genes encoding T6SS-1, MR/P fimbriae, and proteus toxic agglutinin. This Group-specific distribution may explain diversity in pathogenicity of these three species of the genus *Providencia*.

The distribution of antibiotic resistance genes in *Providencia* was determined, and with the exception of common resistance genes, many SDR genes were predominantly distributed in Groups 4 and 5 (*Providencia rettgeri*) and Group 10 (*Providencia stuartii*), which caused a MDR phenotype in *Providencia rettgeri* and *Providencia stuartii*. Furthermore, these MDR genes were mainly derived from class 1 integrons and class 2 integrons rather than the SXT element by HGT events.

Finally, Group-specific genes of the three species of the genus *Providencia* were identified. Strains of *Providencia stuartii* and *Providencia rettgeri* had more mechanisms of material transport and energy metabolism, which reflected a stronger ability to adapt to diverse environments. The presence of the ABC transporters PtgABCP and UgpABCE in strains of *Providencia alcalifaciens* likely contribute to invasion by this species.

In summary, this study enhanced our knowledge of the diversity of pathogenicity, antibiotic resistance, and environmental adaptation of members of the genus *Providencia*. These valuable insights into the large repertoire of antibiotic resistance, pathogenic and environmental adaptation genes, which could have been attained vertically and/or by HGT (mediated by MGEs), may facilitate the development of novel strategies to detect and prevent *Providencia* infection.

## Data Availability Statement

The datasets presented in this study can be found in online repositories. The names of the repository/repositories and accession number(s) can be found in the article/Supplementary Material.

## Author Contributions

CY and ZY conceived the project. SZ and CQ purchased the strains. SZ and YiW prepared the sample DNA for sequencing. CY and YiW performed the bioinformatics analysis. JC and XC organized and saved data. YZ and YuW performed instrument and data maintenance. CY and YiW prepared the manuscript. HC supervised project and reviewed the manuscript. All authors read and approved the final manuscript.

## Conflict of Interest

The authors declare that the research was conducted in the absence of any commercial or financial relationships that could be construed as a potential conflict of interest.

## Footnotes

1 http://www.ncbi.nlm.nih.gov/genome/annotation_prok

## Supplementary Material

Supplementary Figure 1 Structures of CRISPR-Cas systems in *Providencia*. Dark red boxes represented the presence of the gene within a genome, while yellow boxes indicate the absence of the gene.

Supplementary Figure 2 Phylogenetic tree based on core genes of T4SS in *Providencia* **(A)** and *int* genes of integrons and SXT element in *Providencia* **(B)**.

Supplementary Figure 3 Phylogenetic tree based on core genes of T3SS in *Providencia*.

Supplementary Table 1 Genetic characteristics of the strains in the current study.

Supplementary Table 2 Results of pan-genome in *Providencia* after COG annotation, including core, accessory, and unique gene families.

Supplementary Table 3 List of virulence factors in the VFDB database and resistance genes in the CARD database identified in *Providencia* genomes.

Supplementary Table 4 Results of species-specific genes in three *Providencia* species after KEGG annotation.
