# Supplementary figures and images for: Comparative Genomic Analysis Reveals Genetic Mechanisms of the Variety of Pathogenicity, Antibiotic Resistance, and Environmental Adaptation of Providencia Genus
Source: Front Microbiol. 2020 Oct 27;11:572642. doi: 10.3389/fmicb.2020.572642 (PMC7652902; doi:10.3389/fmicb.2020.572642)

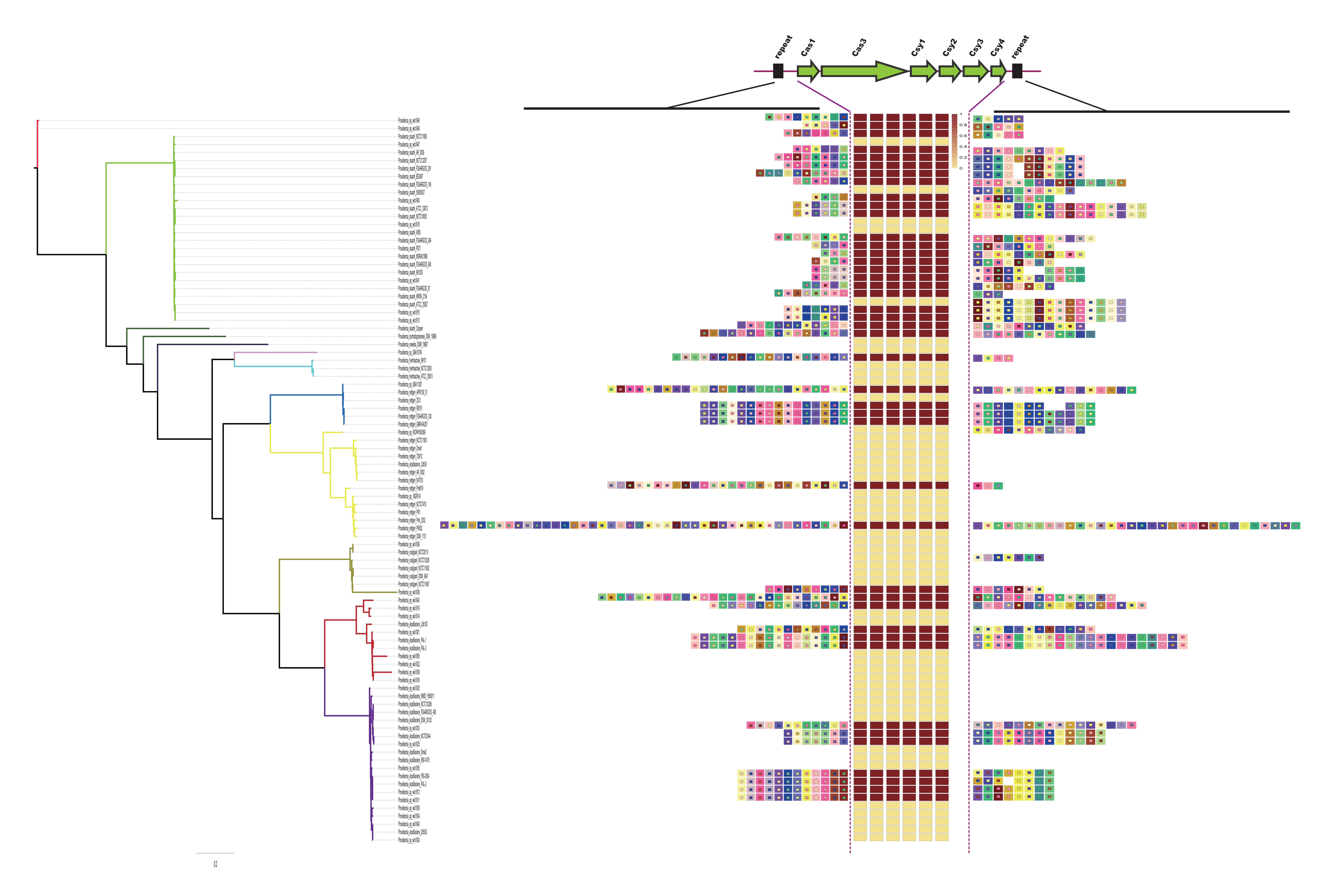

Supplement: Supplementary Figure 1 — Structures of CRISPR-Cas systems in Providencia. Dark red boxes represented the presence of the gene within a genome, while yellow boxes indicate the absence of the gene. [file Image_1.JPEG]

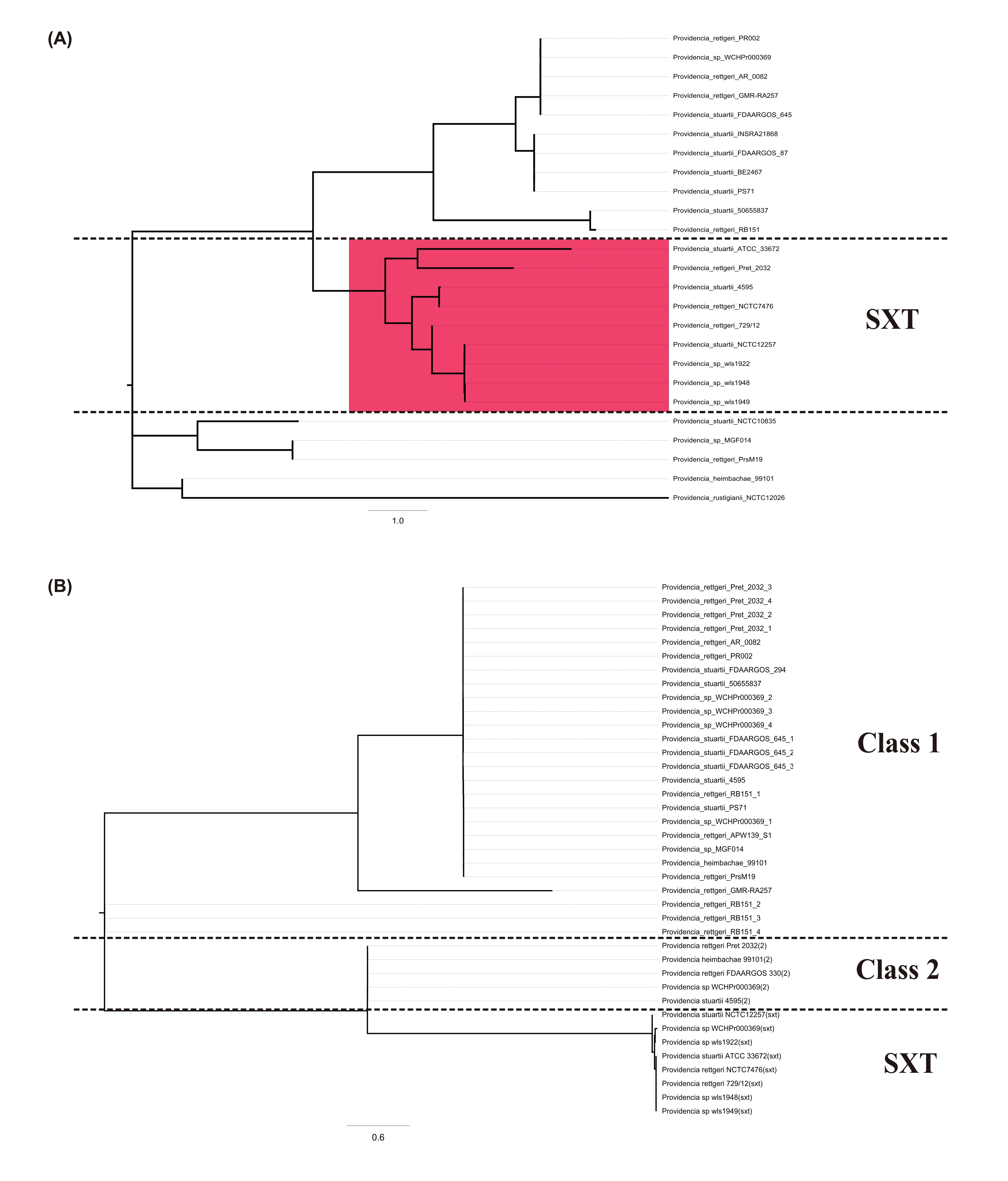

Supplement: Supplementary Figure 2 — Phylogenetic tree based on core genes of T4SS in Providencia (A) and int genes of integrons and SXT element in Providencia (B). [file Image_2.JPEG]

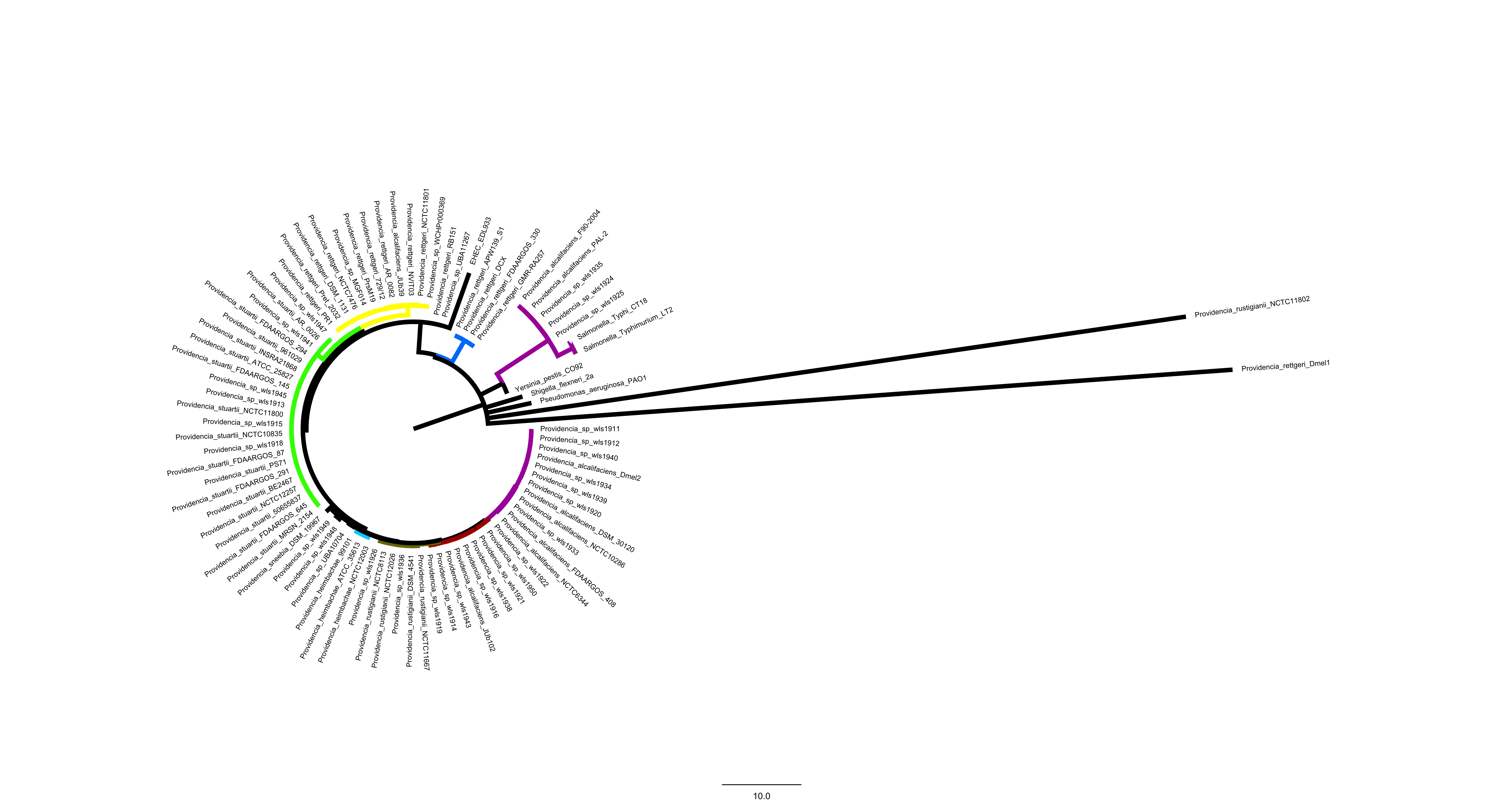

Supplement: Supplementary Figure 3 — Phylogenetic tree based on core genes of T3SS in Providencia. [file Image_3.JPEG]
